# Causal Evidence for Induction of Pessimistic Decision-Making in Primates by the Network of Frontal Cortex and Striosomes

**DOI:** 10.3389/fnins.2021.649167

**Published:** 2021-06-30

**Authors:** Satoko Amemori, Ann M. Graybiel, Ken-ichi Amemori

**Affiliations:** ^1^Institute for the Advanced Study of Human Biology, Kyoto University, Kyoto, Japan; ^2^McGovern Institute for Brain Research and Department of Brain and Cognitive Sciences, Massachusetts Institute of Technology, Cambridge, MA, United States

**Keywords:** anxiety, caudal orbitofrontal cortex, pregenual anterior cingulate cortex, approach-avoidance conflict, monkey, microstimulation, striosome compartment, caudate nucleus

## Abstract

Clinical studies have shown that patients with anxiety disorders exhibited coactivation of limbic cortices and basal ganglia, which together form a large-scale brain network. The mechanisms by which such a large-scale network could induce or modulate anxiety-like states are largely unknown. This article reviews our experimental program in macaques demonstrating a causal involvement of local striatal and frontal cortical sites in inducing pessimistic decision-making that underlies anxiety. Where relevant, we related these findings to the wider literature. To identify such sites, we have made a series of methodologic advances, including the combination of causal evidence for behavioral modification of pessimistic decisions with viral tracing methods. Critically, we introduced a version of the classic approach-avoidance (Ap-Av) conflict task, modified for use in non-human primates. We performed microstimulation of limbic-related cortical regions and the striatum, focusing on the pregenual anterior cingulate cortex (pACC), the caudal orbitofrontal cortex (cOFC), and the caudate nucleus (CN). Microstimulation of localized sites within these regions induced pessimistic decision-making by the monkeys, supporting the idea that the focal activation of these regions could induce an anxiety-like state, which subsequently influences decision-making. We further performed combined microstimulation and tract-tracing experiments by injecting anterograde viral tracers into focal regions, at which microstimulation induced increased avoidance. We found that effective stimulation sites in both pACC and cOFC zones projected preferentially to striosomes in the anterior striatum. Experiments in rodents have shown that the striosomes in the anterior striatum project directly to the dopamine-containing cells in the substantia nigra, and we have found evidence for a functional connection between striosomes and the lateral habenular region in which responses to reward are inhibitory. We present here further evidence for network interactions: we show that the pACC and cOFC project to common structures, including not only the anterior parts of the striosome compartment but also the tail of the CN, the subgenual ACC, the amygdala, and the thalamus. Together, our findings suggest that networks having pACC and cOFC as nodes share similar features in their connectivity patterns. We here hypothesize, based on these results, that the brain sites related to pessimistic judgment are mediated by a large-scale brain network that regulates dopaminergic functions and includes striosomes and striosome-projecting cortical regions.

## Introduction

As a base for our work, we have used the introduction of approach-avoidance conflict to characterize decision-making under an anxiety-like state in non-human primates. This strategy is grounded on the long-standing clinical assessment of behavior by the use of so-called approach-avoidance (Ap-Av) tasks in which the key design is the confrontation of both reward and punishment at the same time. In human subjects, the choices of whether to accept or to reject such mixed offers can elicit psychological conflict and levels of acceptance (Ap, approach) or rejection (Av, avoidance) of behavioral responses, all of which give clues to the presence or absence of states of anxiety or depression (Dickson and MacLeod, 2004; Aupperle and Paulus, 2010; Trew, 2011; Struijs et al., 2017).

**FIGURE 1:**
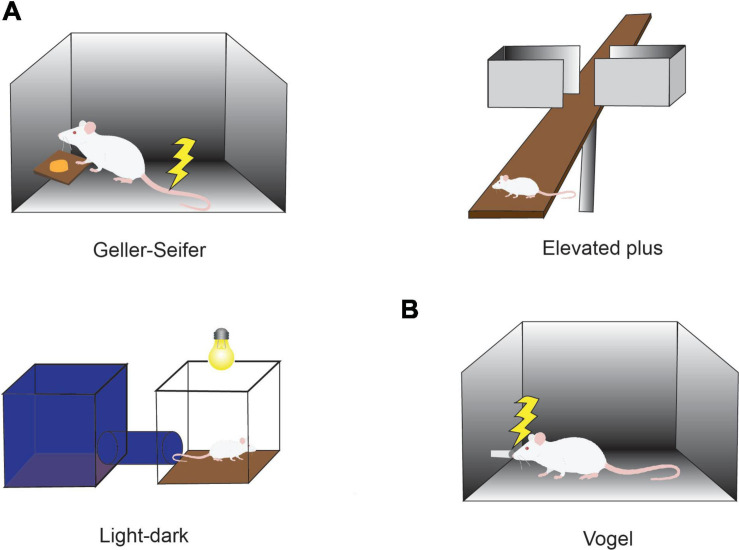
Conflict tasks used to quantify the effects of anxiolytics. **(A)** Conflict task used in preclinical studies of anxiolytics. In the Geller-Seifer conflict test (left), the rat was trained to push the lever under conflict conditions in which reward could be obtained by pushing the lever, but it caused electric shock at the same time. The elevated plus-maze test (right) and the light-dark box test (bottom left) are often used because they are simple and can examine the behaviors under conflict situations between the desire to approach a new condition and the need to avoid anxiety factors such as high place and strong light. The effects of anxiolytics have been measured by observing the behaviors in these tasks. **(B)** Vogel conflict test. Changes in drinking frequency were measured in the Vogel conflict test. Rats frequently lick when they do not receive an electric shock, but the frequency decreases with an increase in the frequency of electric shock. Recovery of drinking frequency was observed depending on the concentration of benzodiazepine (Chlordiazepoxide) even under the condition that an electric shock was simultaneously delivered.

Behavioral Ap-Av conflict tasks were introduced with the goal of quantifying such states (Vogel et al., 1971; Chen and Bargh, 1999), in light of evidence from many studies suggesting that anxiety disorder and characteristics of anxiety can be estimated by the use of Ap-Av conflict tasks (Figure 1; Beck and Clark, 1997; Heuer et al., 2007). For example, it has been reported that anxious humans have a strong desire to avoid looking at the image of an angry face (Roelofs et al., 2010) and tend to exhibit a notable avoidance reaction to crowds (Lange et al., 2008). These changes in frequencies of looking at emotional images and human crowds would become behaviorally apparent, especially in a conflict condition, in which approach and avoidance motivations were in competition. According to questionnaire assessments of subjects as having pessimistic and optimistic characteristics, subjects in the anxious groups tend to overestimate possible punishment, exhibiting a desire to choose avoidance (Dickson, 2006), whereas those with depressive characteristics tend to underestimate future rewards and exhibit less desire to approach (Dickson and MacLeod, 2004). It is now widely accepted that people who easily feel anxiety have a strong desire to avoid, and those who tend toward depression have a weak desire to approach.

## The Primate Anterior Cingulate Cortex and the Decision-Making Under Conflict

Previous studies in macaques have suggested that neurons in the anterior cingulate cortex (ACC) could exhibit the integration of cost and benefit in their responses (Kennerley et al., 2011). The activity of ACC neurons has further been reported to represent motivation to perform the task at hand (Shidara and Richmond, 2002) and a deviation from expected reward (Matsumoto et al., 2007).

**FIGURE 2:**
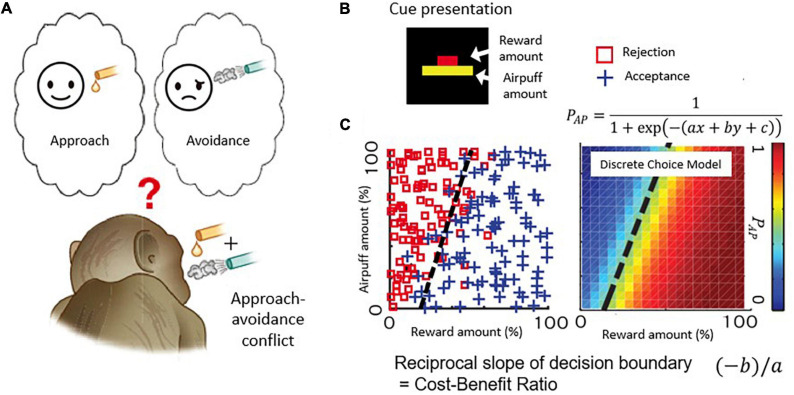
Decision-making task with approach-avoidance conflict. **(A)** When reward and punishment are combined, “motivation for approach” and “motivation for avoidance” become incompatible and conflict occurs. **(B)** Visual stimulus. The length of the upper bar indicates the reward amount, and the length of the lower bar indicates the strength of punishment (air-puff). Macaque monkeys make decisions to accept or reject the combination. **(C)** An example of a monkey’s decision-making. The desire to approach “I want a reward” and the desire to avoid “I want to avoid punishment” make a conflict when reward and punishment are combined. The parameters of the discrete selection model are determined based on the monkey’s actual decision-making pattern.

The ACC thus has been considered as an interconnecting neural element between cognition and emotion. We set out in a series of experiments to ask whether the ACC could be causally involved in conflict decision-making, involving a blending of cognition and emotion. We first developed a version of the Ap-Av conflict decision-making task suitable for the use in non-human primates, with rhesus macaques as our subjects (Figure 2A). With multi-site recording methods (Feingold et al., 2012), we then recorded the activity of many single neurons as the monkeys performed the task and applied microstimulation to determine whether there was a causal influence of the recorded sites on the decision-making of the monkeys (Amemori and Graybiel, 2012).

Figure 2B illustrates the task used in this and with modifications in subsequent work. Each monkey voluntarily started the task by placing its hand on a sensor in front of a joystick, activating the presentation of a compound cue indicating how much reward (red bar) and how much annoying airpuff to the face (yellow bar) the monkey would receive after a decision period in which the monkey viewed the offer. Based on the lengths of the two bars, the monkeys used a joystick-driven cursor to report their decision of whether to approach or to avoid the combination of reward and punishment by moving a cursor to one of two targets that were turned on above and below the compound cue, with random placement. When the monkeys chose the approach target, the offered reward and punishment were given as indicated. When the monkey chose the avoidance target, the monkeys could avoid the combination, but they did receive a small amount of reward to maintain their motivation to continue the task. With this protocol, the choice pattern of the Ap-Av decision-making was usually stable within a single session and was influenced both by the sizes of offered reward and punishment, which were varied independently over ∼100 length steps. Such stability was critical for the experiments. We applied a discrete choice model to estimate how the monkeys judged the value of each choice (i.e., utility function) (Figure 2C; Amemori and Graybiel, 2012), and we characterized them as a linear function consisting of the offered sizes of reward and punishment. Importantly, we also could derive that the slope of the decision boundary between approach and avoidance choices, which in the discrete choice model corresponded to the ratio of internal sensitivities to reward and punishment of the monkeys. Because the reciprocal of the slope of the decision boundary corresponds to the cost-benefit ratio (CBR), we estimated how the monkeys weighted punishment over reward by observing the slope of the decision boundary. Thus, our estimates of the internal decision-making process used by the monkeys were derived from their external actions.

## The pACC and Its Involvement in Pessimistic Decision-Making

**FIGURE 3:**
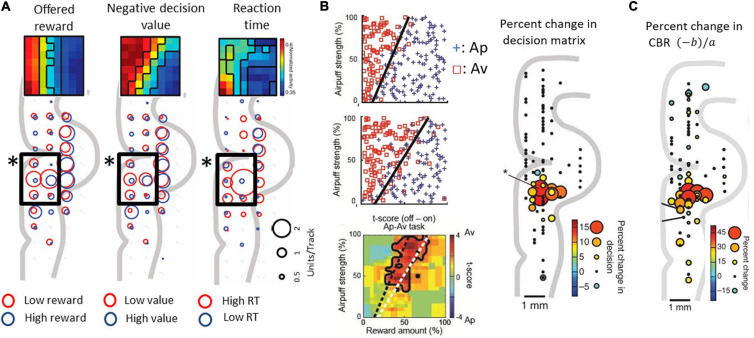
Primate pACC is causally involved in pessimistic decision-making (modified from Amemori and Graybiel, 2012 and Amemori et al., 2018). **(A)** pACC neuronal response. The size of the circle indicates the number of neurons whose activity correlates positively or negatively with the parameter. In the pACC, which is shown by a square frame, neurons that were negatively correlated with presented reward (left), negatively correlated with value (middle), and positively correlated with reaction time (right) were dominant (Fisher’s exact test, *P* < 0.05). The population means of the activity pattern of these neurons are shown in the upper part as a matrix. The horizontal axis is the size of the reward to be presented, and the vertical axis is the air puff strength to be presented. **(B)** Increased avoidance choice by pACC stimulation. (Left figure) The boundary (dashed line) between Ap and Av was changed by delivering the electrical microstimulation in the pACC. Calculation of expected utility with a computational model revealed that pACC microstimulation led to the overestimation of punishment (Right figure) The sites where the small electrical stimulation had an effect on decision-making. **(C)** Increase in CBR due to stimulation (%). The amount of increase was indicated by the size and color of the circle at the stimulation position.

Lowering recording electrodes from the surface of the neocortex through the depth of the medial wall neocortex showed us that many of the encountered neurons were not activated during the performance of the task. However, in and around the anterior cingulate sulcus, we found populations of neurons that responded during the decision period and appeared to encode the relative sizes of the reward and punishment offers, as though they could have reflected Ap-Av decision-making. To investigate the information indicated by the spike activity of these neurons, we performed multiple regression analyses using behavioral and model parameters, including the offered size of a reward, the offered strength of punishment, the utilities for Ap-Av choices, the expected value and the reaction times. We found that neurons with activity positively correlated with the expected value (‘approach neurons’) and others with activity negatively correlated (‘avoidance neurons’) were evenly distributed in the ACC, named as such for convenience for the reader. Our analyses indicated that avoidance neurons were disproportionately distributed in the ventral bank of the cingulate sulcus (Figure 3A), within the pregenual anterior cingulate cortex (pACC), mixed with others classified as approach neurons.

Given that we observed a bias toward avoidance neurons in the pACC ventral bank, we reasoned that this locale within the pACC could be involved in negatively biasing Ap-Av decision-making. We tested this possibility by applying a high-frequency (200 Hz) electrical microstimulation (70–100 μA) during the first second of the 1.5-s stimulus display and by determining whether such focally applied microstimulation could induce changes in the activity of the pACC and the decision-making of the monkeys as they performed the Ap-Av task. They were given 200 trials without pACC microstimulation (no-stim), then 200 trials with the stimulation (stim-on), and a further 200 trials of no-stim. In 13 out of 93 sites, the monkey’s decision significantly changed between trials before and during the microstimulation. The changes were increases in avoidance behavior. All of these ‘effective sites’ were clustered together in the ventral bank of the anterior cingulate sulcus, where we had found the neural activity related to decision-making (Figure 3B). Stimulating at other tested sites was ineffective: critically, we found no effects of the microstimulation on the control Ap-Ap task that was carried along throughout the experiments.

We further found that the microstimulation induced a decrease in the slope of the decision boundaries, which corresponded to an increase in the CBR. The microstimulation was applied in each trial of the stimulation blocks exhibiting such increases in the CBR. This increase indicates that the internal relative value of reward and punishment had gradually changed, leading to pessimistic value judgments related to over-estimation of the forthcoming punishment relative to reward (Figure 3C). Reasoning that, if this were true, anxiolytic treatment might reduce the negative bias, we administered the anxiolytic, diazepam, prior to the stim-on trials. This treatment fully abolished excessive avoidance choices. Other effects of stimulation included an increase in reaction time, which was especially observed in high-conflict conditions. However, the stimulation could induce at most barely detectable change in autonomic signs such as pupil size and skin conductance, suggesting that the microstimulation levels that we applied were too weak to induce strong fear or pain.

These results suggest that the induced abnormal activity of in the pACC effective region could selectively influence the process of integrating reward and punishment normally accomplished by pACC-containing network activity. Because the microstimulation primarily induced an overestimation of punishment, we considered that activity in the particular pACC locale identified could lead to pessimistic decision-making resembling an aspect of anxiety, here deduced directly by the decision-making of the monkeys. The failure of any influence being found on the choice behavior by stimulating during the Ap-Ap task, in which both options were good, is compatible with this conclusion. The pessimistic decision-making that we induced was recovered by diazepam administration and could be one aspect of anxiety. We have recently demonstrated congruent neural correlations of the Ap-Av conflict and aversive responsiveness in the pACC between human and macaque subjects (Ironside et al., 2020), suggesting that the neural mechanisms observed in macaques could also exist in humans.

## The pACC-Striosomal Circuit and Its Involvement in Pessimistic Decision-Making

**FIGURE 4:**
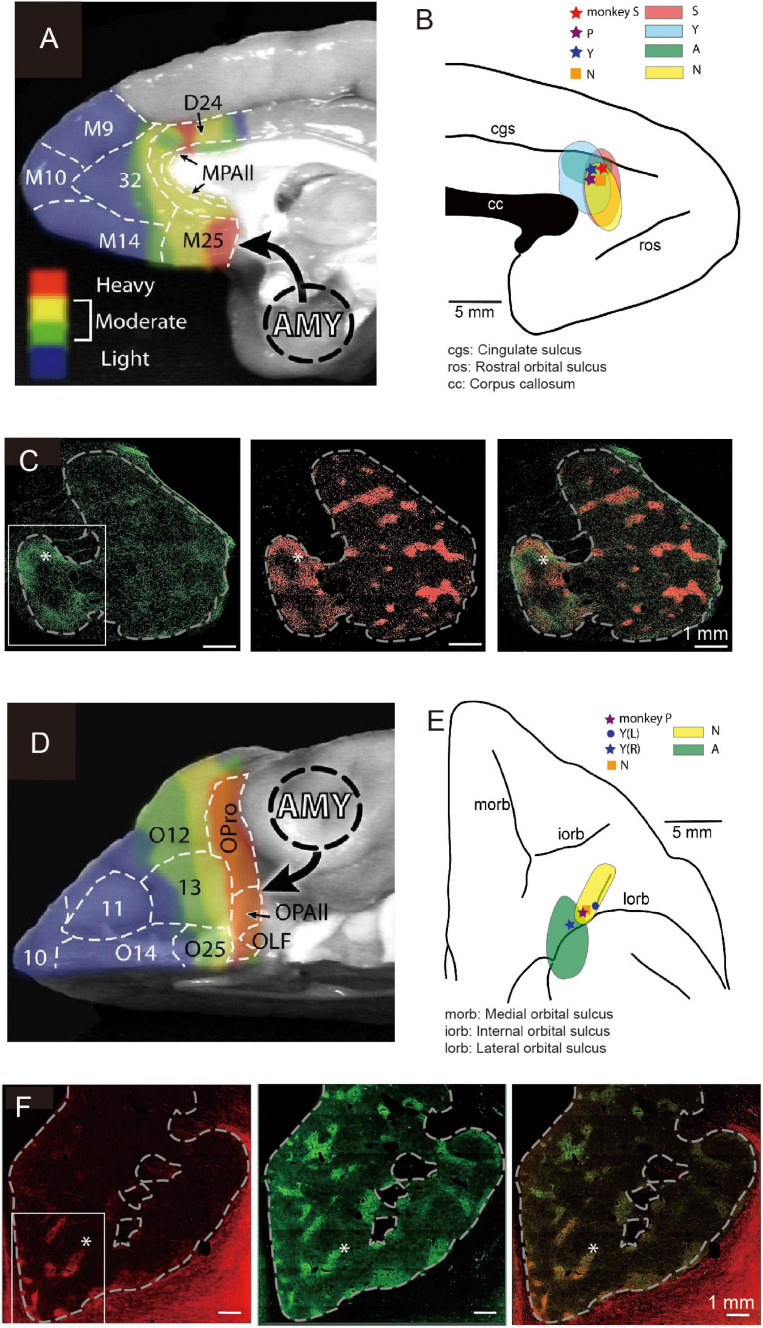
Local sites of primate pACC and cOFC that project to the striosome compartment of the striatum causally involved in pessimistic decision-making (Modified from Amemori et al., 2020). **(A)** Primate pACC and sACC receive direct projection from the amygdala (Adapted from Ghashghaei et al., 2007). **(B)** Viral tracer was injected into a local region of the pACC (left) where avoidance behaviors were induced by electrical stimulation. **(C)** (Left) Projection to the striatum from the pACC local site that had a stimulating effect. (Middle) Striosomes shown by KChIP1 staining. (Right) Overlay between left and middle images. **(D)** Primate cOFC receives direct projection from the amygdala (AMY) (Adapted from Ghashghaei et al., 2007). **(E)** Viral tracer was injected into a local region where avoidance behaviors were induced by electrical stimulation of cOFC. **(F)** (Left) Projection to the striatum from the cOFC local site that had a stimulating effect. (Middle) Striosomes shown by KChIP1 staining. (Right) Overlay between left and middle images. The projection to striosomes was dominant.

The primate pACC is interconnected with multiple regions within the prefrontal cortex (Pandya et al., 1981) and sends outputs to multiple subcortical structures. In particular, the pACC, as well as the subgenual anterior cingulate cortex (sACC) and the caudal region of the orbitofrontal cortex (cOFC), are, in particular, limbic cortical regions that have direct inputs from the amygdala (Ghashghaei et al., 2007; Figures 4A,D). The sACC, just ventral to the pACC, projects strongly to the ventral striatum (VS), prominently to the nucleus accumbens (NAcc) (Ferry et al., 2000; Haber et al., 2006; Haber and Knutson, 2010). The pACC and cOFC have preferential projections to the striosome compartment in the striatum, especially to the anterior striosomes of the caudate nucleus (CN) and adjoining rostral part of the putamen (Eblen and Graybiel, 1995). But nothing was known about the functional importance of this corticostriatal circuit.

We designed an experiment to address this issue. Having found the small locale in the ventral bank of the cingulate sulcus, we again mapped the region while the monkeys performed the Ap-Av decision-making task to find sites at which microstimulation induced an increase in avoidance choices. In other experimental sessions, we further performed microstimulation experiments for functional mapping of the cOFC. We then injected anterograde viral tracers at the sites effective in changing the monkeys’ decision-making and traced the efferent projections of the sites (Figures 4B,E). We found that the effective sites have major projections to the striatum, preferential to the striosome compartment (Figures 4C,F). Although the predominant projection from the pACC and cOFC to striosomes is striking, the viral labeling in the anterior CN and putamen was not exclusively located in striosomes. The pACC-striatum pathway substantially also includes projections to the matrix compartment (Ragsdale and Graybiel, 1981; Eblen and Graybiel, 1995; Sgambato-Faure et al., 2016; Amemori et al., 2020). The zone of most preferential targeting of striosomes appears to be quite localized, as shown not only in our own work (Eblen and Graybiel, 1995; Amemori et al., 2020) but also in direct comparisons of cases with nearby injection sites, such as shown in the anatomical work of (Schmahmann and Pandya, 2009). We note, however, that our control injection in the cingulate motor area did not detectably label striosomes. These findings favor a working conclusion that local circuits within pACC and cOFC that project to striosomes could be causally involved in biasing decision-making induced by their microstimulation. Because this negative bias with microstimulation of the pACC and cOFC was ameliorated by anxiolytics, these results lead to the hypothesis that one of the major outputs of limbic cortices related to the induction of pessimistic value judgment, compatible with an anxiety-like state, is the striosome compartment of the striatum (Amemori et al., 2020).

**FIGURE 5:**
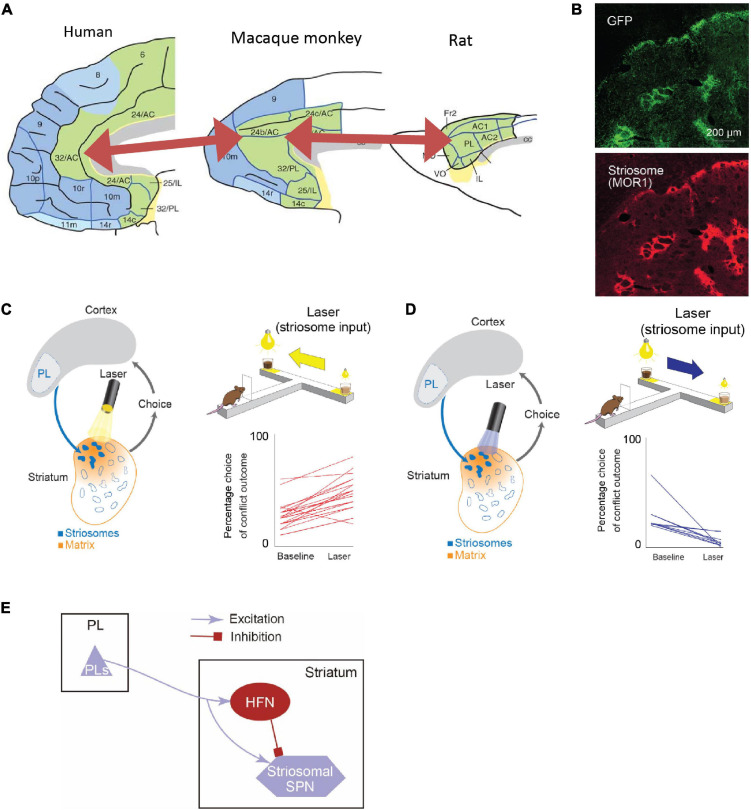
The rodent PL-striosomal pathway is causally involved in pessimistic decision-making (Adapted from Friedman et al., 2015). **(A)** Comparison of human, macaque, and rat limbic cortices. In the macaque areas 24/32 and rat prelimbic cortex (PL), the projection to striosomes is predominant. The macaque areas 24/32 are considered to be homologous to the human areas 24/32 (Adapted from Wise, 2008). **(B)** GFP-labeled rat PL striatal projections (top) and striosomes identified by MOR staining (bottom). **(C)** Pathway-selective inhibition of PFC-PL-striosomal pathway by halorhodopsin increased selection of chocolate milk under strong light (high-cost, high-reward option). **(D)** Pathway-selective excitation of PFC-PL-striosomal pathway by channelrhodopsin increased selection of diluted chocolate milk under dim light. **(E)** Schematic diagram of PFC-PL-striosomal circuit. In conflict task, optogenetic inhibition of the PFC-PL pathway **(C)** released activity in the striosomes via the inhibition of HFNs during the time when the animals greatly increased their approaches to high-cost, high-reward goals. By contrast, optogenetic excitation of this pathway **(D)** tuned off striosomes via the activation of HFNs during the time when the animals decreased their approaches to such goals.

Anatomical work has shown that the connectivity from the sACC to the VS exhibits a common pattern between rodents and primates (Heilbronner et al., 2016; Figure 5A). Contrarily, the projection pattern from the pACC to the striatum was reported to be complicated. We searched for a brain region in rodents that exhibited a similar projection pattern to that of the pACC region projecting preferentially to striosomes (Friedman et al., 2015), and we found, and confirmed, that the anterior part of the rat’s prelimbic cortex (PL), which to be conservative in nomenclature, we called prefrontal cortex (PFC)-PL, projects preferentially to striosomes, as does the small pACC region in macaques (Figure 5B). Taking advantage of this selective projection pattern (Gerfen, 1984; Donoghue and Herkenham, 1986; Friedman et al., 2015), we aimed to identify functions of the cortico-striosomal circuit using optogenetics. We performed selective inhibition of the PFC-PL cortico-striosome pathway using local halorhodopsin injection applied to the terminals of the corticostriatal fibers estimated to be in striosomes in the anteromedial caudoputamen. This inhibition could strongly increase the frequency of approaching a high-conflict offer, here, pure chocolate milk combined with bright light shined on a reward well of a T-maze in which the offer on the other side was low-light but diluted chocolate milk (Figure 5C). Thus, inhibiting the PFC-PL-striosomal pathway prompted the rats to run to the “bad” offer. By contrast, selective activation of the pathway by channelrhodopsin increased the frequency of avoiding a high-conflict Ap-Av offer while not affecting behavior exhibited in four other types of compound-offer tasks (Figure 5D). These results suggest that the activity of the cortico-striosomal circuit could be causally involved in modulating valuation estimation changes in conflict contexts (Friedman et al., 2015).

Further experiments strongly implicated a local intrastriatal circuit as being the switching mechanism (Friedman et al., 2015, 2017, 2020). The circuit, to our current estimation, controls by fine-resolution temporal dynamics the relative time of arrival of cortical commands to the striosomal projection neurons and their inputs from the implicated interneurons, also innervated by the cortical circuit. The microcircuit that we identified likely implicates the high-firing neurons (HFNs) that we were studying in these experiments (Figure 5E).

We further found that this PFC-PL-striosomal pathway is vulnerable to mild chronic stress, producing increased running to the high-conflict side of the maze and that rescue from the behavioral effects of the dysfunction could be affected by manipulating the local intrastriatal circuit, including putative HFNs. Thus again, abnormal value judgment under conditions of Ap-Av conflict occurred, and now could be causally associated with the cortico-striosomal system and its local circuit modulation (Friedman et al., 2017). New work (Friedman et al., 2020) has now extended the functional study of this pathway to address its potential role in value-based learning involving valence discrimination and in the ability to engage in tasks. With chemogenetics, our group found that a motivational state characterized by the level of task engagement could be causally influenced by levels of striosome-predominant activity in contrast to a lack of such an effect by matrix-predominant activity. The striosomes and their network connectivity with cortical and other subcortical regions thus have a broad potential significance to behavior and likely can modulate value-based decision-making and action in conflict situations so as to favor, or even to induce and control, anxiety-like states.

## Functional Contrasts Between the pACC and the Striatum

**FIGURE 6:**
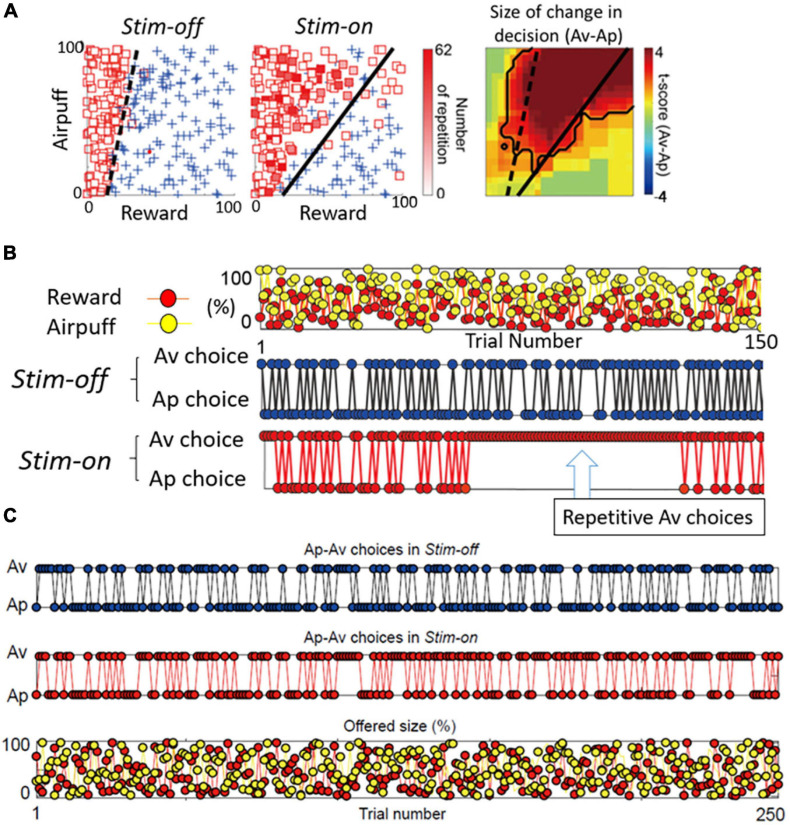
Primate CN is causally involved in persistent pessimistic decision-making. **(A)** An example of a monkey’s decision-making. Changes in decision-making patterns (avoidance: square, approach: cross) due to microstimulation (left: before stimulation, middle: during stimulation). Blue cross indicates approach, and red square indicates avoidance. The color of each square is filled in according to the number of repeated avoidances. It can be seen that there were many repeated avoidances during microstimulation. (Right) Local stimulation of the striatum significantly increased avoidance decisions (Fisher’s exact test, *P* < 0.05). **(B)** CN stimulation caused an abnormal continuous avoidance choice. (Top) The same reward (red circle) and punishment (yellow circle) sequences were presented before and during stimulation. (Middle) The decision to approach/avoidance decisions are shown in the order of trial numbers. (Bottom) A sequence of abnormal repeated avoidance choice was found during stimulation. **(C)** pACC stimulation did not cause a continuous avoidance choice. Sequences of the Ap-Av decisions in the Stim-off (top) and in the Stim-on (middle) blocks, and the sequence of the reward (red circles) and airpuff (yellow circles) sizes (bottom) (Adapted from Amemori et al., 2018).

If both the pACC and striatum are causally involved in inducing pessimistic decision-making, what distinguishes their contributions? From work in our laboratory on mice and rats, glimpses of such differences are apparent. But this issue is much more difficult to address in non-human primates, as we do not yet have adequate genetic means to label differentially the striosomes and the matrix with functional markers. As a first step, however, we performed microstimulation of the CN of macaques as they performed Ap-Av tasks, and we compared the effects of this stimulation with the effects of pACC microstimulation (Amemori et al., 2018). Out of 112 CN sites, the microstimulation of 13 sites (12%) induced an increase in approach, and we designated them as “approach sites.” This result, and the concomitant decrease in the estimated CBR indicated by the monkey’s behavior, suggested that the stimulation could have induced optimistic value judgment. At 25 sites (22%), CN microstimulation induced an increase in avoidance, and we designated them as “avoidance sites” (Figure 6A). Effective stimulation also significantly increased the reaction time (RT) and pupil size, supporting the hypothesis that the monkeys might have been motivationally influenced. As the behavior indicated an increase in the estimated CBR and the administration of anxiolytics ameliorated the negative bias, we considered that these effective-site stimulations could have induced pessimistic value judgment, compatible with a pessimistic decision-making underlying anxiety. The pessimistic decision-making induced by the CN stimulation did not recover as soon as we stopped the stimulation.

By examining in detail the trial-by-trial behavioral effects of the CN microstimulation, we found that at the effective avoidance sites, the stimulation additionally produced a significant consecutive repetition of avoidance choices (Figure 6B). Those were not observed by pACC microstimulation (Figure 6C). These behavioral changes strongly suggest that the CN microstimulation evoked a persistent repetition of negative decision-making. By virtue of the effective avoidance-site CN stimulation, the monkey could not flexibly switch between optimistic and pessimistic decision-making. These findings favor the conclusion that the induced abnormal activity of CN could be an underlying basis for these persistent pessimistic decisions.

**FIGURE 7:**
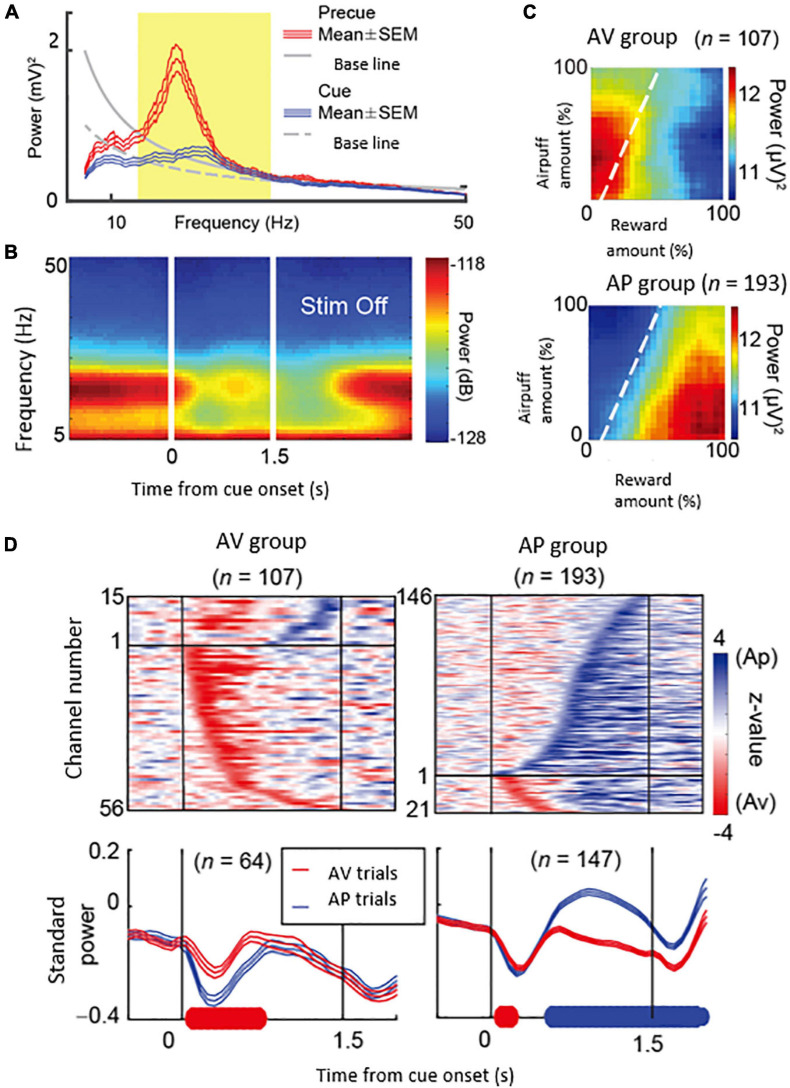
Beta oscillations in the striatal local field potentials (LFPs) encode approach-avoidance decision (Adapted from Amemori et al., 2018). **(A)** Spectral intensity in precue (red) and cue (blue) periods and baseline (gray). Yellow shading represents the range of beta oscillations. **(B)** A group of beta oscillations responding to the Ap-Av task. Spectrogram in which time 0 indicates the start of cue presentation. **(C)** Beta oscillations related to emotion during cue period. Av group (top) appeared in avoidance decisions. Ap group (bottom) appeared in approach decisions. The X-axis indicates the amount of rewards presented (length of the red bar) and the Y-axis indicates the intensity of punishment presented (length of the yellow bar). The color changes indicate the strength (power) of beta oscillations. The dotted line shows the boundary of Ap-Av decision-making. **(D)** Onset of beta response in cue period. Top: Time change of information representation (power difference, *z*-value) for Ap (blue) and Av (red). Ap group (right) and Av group (left). Bottom: Power of Beta wave. Solid bold line indicates the time period that shows significant differences between Ap and Av trials (red: Av > Ap, blue: Ap > Av, *t*-test). Av Group expressed value judgment earlier in the cue period.

What could be the neural mechanism of CN that underlies these persistent pessimistic decisions? The answer to this question is yet to be determined; we did, however, make a further set of observations that could well bear on the answer. During the CN microstimulation experiments, we simultaneously recorded neural activity from chronically implanted multi-electrode arrays distributed over 1 mm of tissue and then analyzed the local field potentials recorded by these. We found that beta-band oscillations during the decision period could, in some instances, exhibit activity predictive of the upcoming avoidance decisions (Figure 7). The population activity of the beta-band responses further showed that the magnitude of the beta oscillations was correlated with the level of avoidance behavior induced by the CN microstimulation. These results suggest that the CN beta oscillations could underlie the abnormally persistent pessimistic decisions, and if we accept this behavior as a proxy indicating an internal state of anxiety or negative engagement, then these results point to beta-band activity as a concomitant of such state change. We cannot make statements about the compartmental identity of the effective sites; this is clearly a crucial issue to resolve. But we note that beta-band activity is, at the relatively long time-frames resolved here (Schwerdt et al., 2020), is negatively correlated with dopamine release (Kühn et al., 2006; Jenkinson and Brown, 2011).

Compared to the optogenetic experiments so readily feasible in rodents, the microstimulation experiments, still a primary mode of manipulation in macaques, have clear methodological limitations. The electrical stimulation can influence the neurons around the site we stimulated, as well as the passing fibers irrelevant to, or different from, the neuronal circuit that we targeted. Therefore, in the microstimulation experiments, we recorded neuronal activity and confirmed positive correlations between neuronal features and the stimulation effects (Amemori et al., 2018). We could not, however, exclude the possibility that the stimulation influenced off-target, including distant, sites connected with the stimulation sites. As with other manipulation experiments, these experiments do not adequately mimic endogenously generated activity states influenced by internal or external occurrences.

## Continuing Questions About the Functions of Striosomes

The striatum is histologically classified into two compartments, the striosome (also called a patch) and matrix (Graybiel and Ragsdale, 1978; Brimblecombe and Cragg, 2017). The striosome compartment consists of three-dimensional labyrinth-like structures (Mikula et al., 2009) and winds as labyrinthine extensions through the much larger matrix compartment, which makes up about 10–15% of the striatum (Desban et al., 1993). The striosomes are characterized by their chemical and molecular expression patterns relative to those of the matrix compartment (Graybiel, 1990; Crittenden and Graybiel, 2011). For example, striosomes exhibit high-level expressions of μ-opioid receptors (MORs), substance P, met-enkephalin (Graybiel and Chesselet, 1984), limbic system-associated membrane protein (Prensa et al., 1999), and other substances as well. In macaque monkeys, striosomes are characterized by immunohistochemistry with voltage-gated potassium channel interacting protein 1 (KChIP1) (Mikula et al., 2009). The matrix is enriched in calbindin, somatostatin, enkephalin, acetylcholinesterase and acetylcholine (Graybiel and Ragsdale, 1978; Graybiel, 1984; Graybiel et al., 1986).

Recent studies suggest that spiny projection neurons (SPNs) in striosomes and those in the matrix have few direct synaptic interactions (Miura et al., 2008; Lopez-Huerta et al., 2016), with some exceptions in their borders (Bolam et al., 1988; Burke et al., 2017). It is usually considered that the SPNs of the two compartments have distinctive and independent functions (Lopez-Huerta et al., 2016), but there is clear evidence that cholinergic interneurons (ChIs) located in the matrix can extend fine, presumably axonal, fibers into striosomes (Graybiel et al., 1986; Crittenden et al., 2017). For the downstream of these two compartments, a clear difference in the projection pathways to the substantia nigra from striosomes and matrix has been reported in rodents and felines. An outstanding example is that the striosomes have projections to the dopamine-containing substantia nigra pars compacta (SNc) (e.g., Jimenez-Castellanos and Graybiel, 1987; Fujiyama et al., 2011; Crittenden et al., 2016; McGregor et al., 2019; Evans et al., 2020; Matsushima and Graybiel, 2020).

The classical view of the striatonigral system is that it is inhibitory, given that the SPNs are GABAergic and project without synaptic interruption to cells in the substantia nigra, whether it is non-dopaminergic pars reticulata (SNr) or the dopamine-rich SNc. Thus the function of the striosome-SNc pathway would be to suppress dopamine (DA) cells. The response of DA cells to striatal electrical stimulation is predominantly suppressed (Brazhnik et al., 2008). Recent studies used genetically modified mice as a new tool for identifying the projection pattern of striosomal SPNs. In slice preparations, in which striosomal input fibers and DA-containing neurons could be identified, activation of the striosomal fibers produced inhibition of the DA-containing neurons (McGregor et al., 2019). However, this inhibitory phase can be followed by a prolonged rebound excitation (Evans et al., 2020), suggesting that the response cannot always be categorized as an inhibition.

In addition to the direct potentially inhibitory projection to the DA cells, many striosomal SPNs also project to a specialized part of the pallidum (GPh), and this part in turn projects to the lateral habenula (LHb) (Matsumoto et al., 2007; Hong and Hikosaka, 2008; Stephenson-Jones et al., 2016; Hong et al., 2019). The GPh input fibers have both glutamate and GABA, so that its remarkable excitation of the LHb could also have an inhibitory component (Hong and Hikosaka, 2008; Shabel et al., 2012). The LHb, in turn, is thought to suppress DA cells via excitatory projections onto the rostral medial tegmental nucleus (RMTg) (Jhou et al., 2009; Hong et al., 2011). With these facts at hand, it is possible that striosomes could inhibit and excite DA-containing neurons. Much more study would be needed to determine whether and how striosomal SPNs could excite DA neurons. These striosomal access routes to the DA-containing neurons are conserved across estimated evolutionary time; the projections from putative striosome and matrix compartments to the globus pallidus (GP) are markedly separated in lamprey, forming separate nuclei (Stephenson-Jones et al., 2013). Even in rodents and primates, the pathways from the striosomes and matrix are clearly independent (Stephenson-Jones et al., 2016; Hong et al., 2019). We have shown that electrical stimulation in or very near striosomes in the macaque striatum evokes responses in electrophysiologically identified LHb neurons (Hong et al., 2019).

## Computational Modeling Characterizing Striosomal Function

From the computational perspective, the distinct projection patterns from striosomes and matrix to the SNc (Jimenez-Castellanos and Graybiel, 1987) have been likened to the parallel network structure of the actor-critic (AC) model of reinforcement learning (RL) theory. The AC model consists of two parallel circuits: one for the ‘actor’ and the other for the ‘critic.’ For each state (s*_*t*_*), the ‘critic’ calculates the reward prediction error. The reward prediction error can be derived by the temporal difference (TD) error, which corresponds to the difference between the prediction at time *t* [V(s*_*t*_*)] and the prediction at the next time point *t+1* after knowing how much the agent could obtain rewards [r*_*t*__+__1_* + γV(s*_*t*__+__1_*)]. By adjusting V(s*_*t*_*) to r*_*t*__+__1_* + γV(s*_*t*__+__1_*) for every time *t*, the agent’s reward prediction eventually becomes accurate even at the beginning of the trial (*t* = 0). The ‘actor’ of the AC model, on the other hand, calculates the action value [Q(s*_*t*_*, a*_*t*_*)], which is used for the selection of actions (a*_*t*_*). The reward prediction error can be used to update the action value, reinforcing the action that would eventually lead to acquiring rewards. In addition to the empirical success of the AC model (Mnih et al., 2016), the convergence of learning with some theoretical settings has also been proven. In the striatal circuit model, striosomes are key by corresponding to critics, and the matrix corresponds to the different actors (Houk and Adams, 1995; Doya et al., 2002; Joel et al., 2002; Takahashi et al., 2008).

The parallel structure of the basal ganglia circuitry can be modeled by modular RL architectures (Doya et al., 2002; Amemori et al., 2011). In the model that we proposed (Amemori et al., 2011), we assumed that the activity of the striatal ChIs calculates a responsibility signal that represents the degree of suitability of the corresponding module (a striosome and surrounding matrix components) to the environment. The dysfunction of the CN could be modeled by the impairment of the responsibility signal in the modular RL architecture, leading to an impairment in rapid adaptation to the change in the environment. In our primate experiment, the microstimulation of the CN caused repeated abnormal avoidance choices (Figure 6) (Amemori et al., 2018). Our model suggests that behavioral persistence and repetitive behaviors, such as obsessive-compulsive disorder, could be evoked by abnormal striosome-matrix balance or dysfunction of intervening ChIs. In concurrence with this view, an abnormal striosome-matrix imbalance can be produced by chronic exposure to psychomotor stimulants. As judged by early-gene detection methods, the excessive striosomal activity relative to that of the matrix could be induced by the challenge exposure, in which rodents and non-human primates showed stereotypic and repetitive behavior (Canales and Graybiel, 2000; Saka et al., 2004). When the striatal ChIs were selectively ablated (along with somatostatin interneurons, due to a toxin affecting substance P receptors expressed by them), chronic psychomotor stimulant exposure no longer produced striosome-matrix imbalance (Saka et al., 2002). ChIs are involved in the regulation of the dopaminergic release in the striatum (Rice and Cragg, 2004; Cragg, 2006), differentially targeting striosomes and matrix. In the modular RL model, the learning ought to happen specifically at the selected module but ought to be suppressed at unselected modules. The striatal ChIs are sparsely distributed in the striatum, and each ChI is posited to have an individual influence on each striatal module. The individual influence of ChIs on the dopaminergic release might give support to accomplish the selection of modular learning.

## Long-Distance Interconnections of Limbic Regions Involved in Inducing Pessimistic Decision-Making

The pACC and cOFC share two important features, as shown by our experimental characterization of these cortical regions (Figure 4). First, microstimulation of both the pACC (Figure 4B) and the cOFC (Figure 4E) induced an increase in avoidance choices in the Ap-Av task, suggesting that these cortical regions directly or indirectly produce the causal chain involved in the generation of a symptom often expressed in an anxiety-like state. Second, with viral tracers injected into behaviorally identified avoidance hot-spots in the pACC and in the cOFC, we found that both effective-site locales exhibited enriched projections to the striosome compartment in the anterior striatum (Figures 4C,F), and we observed, as discussed below, cortico-amygdala projections in these behaviorally guided tracer injection experiments as well (Amemori et al., 2020, and present report). The connectivity of these regions with the amygdala confirms and extends the series of previous anatomical studies. The primate amygdala has been shown to have reciprocal connections with the pACC (Figure 4A) and cOFC (Figure 4D) (Ghashghaei et al., 2007). Thus, the two different and distant cortical regions, pACC and cOFC, could similarly interact with the amygdala, suggesting that they might have functional interactions. We therefore re-analyzed our anatomical dataset from experiments with tracing viral labeling from pACC and cOFC avoidance hot-spots reported in our previous article (Amemori et al., 2020).

After we determined sites at which microstimulation effectively induced an increase in avoidance decisions (star and circle marks in Figures 4B,E) in monkeys (monkeys S, P, and Y for pACC, and monkeys P and Y for cOFC), we injected anterograde virus tracer into the site to label the projections (monkeys S, Y, N, and A for pACC, and monkeys Y, N, and A for cOFC). A total 1 μl of AAV-DJ-CMV-mCherry (genomic titer: 2.10E+14 vg/ml; infectious titer: 3.30E+10 IU/ml) and a total of 0.9 μl of AAV-DJ-CMV-hrGFP (genomic titer: 1.10E+14 vg/ml; infectious titer: 2.50E+09 IU/ml; Stanford Vector Core), respectively, were pressure-injected into the pACC, and the cOFC sites in monkey N. A total 1 μl of AAV-DJ-CMV-mCherry and a total of 1.5 μl of AAV-DJ-CMV-hrGFP were, respectively, injected into the cOFC, and the pACC sites in monkey A. Forty-μm coronal sections of the brain were made and double-stained by the antibodies for hrGFP or RFP and KCHIP1 with immunohistochemical techniques. Then they were imaged by a fluorescent image scanner (TissueFAXS Whole Slide Scanner; TissueGnostics). The virus expressions were observed by eye, and the brain regions were estimated by KCHIP1 images (see the details for methods in Amemori et al., 2020).

We found similar connectivity patterns of these behaviorally identified sites in the pACC and cOFC not only in the striatum, but also more generally in limbic regions. We examined the pACC and cOFC projections in monkeys A and N (Table 1). Those are preliminary data based on observations for two monkeys. The efferent projection targets that we commonly observed in these monkeys included the sACC, the OFC proper, the cOFC, the amygdala, the thalamus, and the tail of the CN (Cdt), in addition to preferential projections to striosomes. The cOFC projection targets that we commonly found in these monkeys included the pACC, the sACC, the OFC proper, the premotor cortex, the temporal cortex, the ventral prefrontal cortex, the anterior insular cortex (AIC), the amygdala, the thalamus, the Cdt, and, again, striosomes.

**TABLE 1:** pACC and cOFC projection sites.

	pACC		cOFC	
	Monkey N(anterior part of area 32)	Monkey A(area 24b)	Monkey N(anterior part of OPro)	Monkey A(OPro)
DPFC		9L, 9M, 8B		
PM		6M, 6DR	6DR	6DR, 6VR
dACC		8/32, 6/32		6/32
pACC	24a, 24c	24c	24a, 24b, 24c	24a, 24b, 24c, 32
sACC	14M, 25	14M, 25	25	14M, 25
OFC	13M, 47o	14O, 47o	47o	13M, 47o
VPFC		44	44	44
Insular Cortex		DI, AI	DI, AI	DI, AI, IPro
cOFC	OPro	OPro		
Temporal Cortex			ST3	TPO
Striatum	CN Putamen Caudate tail	CN Putamen Caudate tail	CN Putamen Caudate tail	CN Putamen Caudate tail
Amygdala	BL	BL	BL	BL
Thalamus	VA, MD	VA	IMD	VA, IMD
Others		Cl	Cl, EC	S2, Cl, EC

*The name and abbreviations of all areas followed in The Rhesus Monkey Brain (Paxinos et al., 2009).*

*DPFC, dorsal prefrontal cortex; PM, premotor cortex; pACC, pregenual anterior cingulate cortex; dACC, dorsal anterior cingulate cortex; OFC, orbitofrontal cortex; VPFC, ventral prefrontal cortex; cOFC, caudal orbitofrontal cortex; S2, secondary somatosensory cortex; DI, dysgranular insular cortex; AI, agranular insular cortex; Cl, claustrum; Ipro, Insular proiso cortex; OPro, orbital proisocortex; TPO, temporal parietooccipital associated area in sts; EC, entorhinal cortex; BL, basolateral amygdaloid nucleus; VA, ventroanterior thalamic nucleus; MD, mediodorsal thalamic nucleus; IMD, intermediodorsal thalamic nucleus; 6M, area 6, medial; 6DR, area 6, dorsorostral part; 6VR, area6, ventrorostral part; ST3, superior temporal sulcus area 3.*

These results demonstrate that two circuitries related to pessimistic decision-making defined by functional efficacy through microstimulation at tracer injection sites in the pACC and cOFC are reciprocally interconnected and further suggest that the pACC and cOFC networks could share similar nodes in their connectivity. The pACC and cOFC projections to the Cdt are particularly interesting as recent reports showed that the Cdt has a crucial role in reflective avoidance (Menegas et al., 2018) and rejection of bad objects (Amita and Hikosaka, 2019). Further, the tracer-labeled projections of the pACC and cOFC reached the sACC, which has been particularly implicated in major depressive disorder and anxiety disorder (Mayberg et al., 2005; Holtzheimer et al., 2017). Both the sACC and pACC have been implicted as contributing to negative emotion, but their functional roles could be different (Wallis et al., 2017). Particularly, the sACC has been implicated in the control of cardiovascular and behavioral arousal responses to threat (Alexander et al., 2020), and the sACC-anterior hippocampus connections were shown to regulate approach-avoidance behavior (Wallis et al., 2019).

Although the pACC and cOFC networks share similar nodes, there are also marked differences in their efferent projection patterns. Remarkably, the cOFC has strong projections to the ventrolateral PFC (vlPFC, area 44), insula cortex, and hippocampus regions such as the entorhinal cortex. In marmoset studies (Clarke et al., 2015), it has been shown that the vlPFC and the OFC are both involved in negative bias in approach-avoidance conflict, but their functional roles could be different. The vlPFC could mediate attentional shifting towards a negative stimulus. The OFC may mediate the consolidation of negative memory by interacting with the hippocampus and amygdala. Another study demonstrated that the lesions of the vlPFC and cOFC in marmoset heightened negative emotional responses (Agustín-Pavón et al., 2012). In summary, pACC and cOFC had similar effects (i.e., an increase in avoidance decisions) with microstimulation and share similar projection patterns. However, it is also likely that they would have different roles in pessimistic decision-making.

## Hypotheses

### A Causal Network for Pessimistic Decision-Making in Primates

**FIGURE 8:**
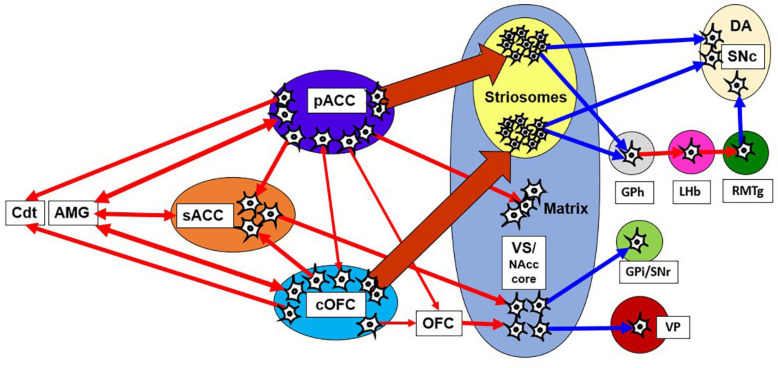
Network of pessimistic decision-making in primates. The network consists of the pACC and cOFC that have interconnections and could reciprocally interact with the amygdala and sACC. The pACC and cOFC send output signals to the Cdt, thalamus and striosome compartment of the striatum, which in turn could modulate dopaminergic signals directly and indirectly. Red line indicates an excitatory connection, and a blue line indicates inhibitory connection. The pACC and cOFC send signals preferentially to striosomes in the anterior striatum. The VS-VP pathway is causally involved in anxious behaviors such as stereotypies. AMG, amygdala; GPi, internal globus pallidus.

Meta-analyses of human fMRI studies have suggested that patients with anxiety disorders consistently showed greater activity than matched comparison subjects in the amygdala and insula, structures linked to negative emotional responses (Etkin and Wager, 2007). Neuroimaging studies in humans have demonstrated that multiple brain regions, including the dorsolateral PFC (dlPFC), ACC, striatum, OFC, amygdala, and anterior insula, underlie approach-avoidance decisions or other anxiety-related behaviors (Grupe and Nitschke, 2013; Aupperle et al., 2015). In non-human primates with anxious temperament (AT), which corresponds to the monkey’s innate pessimistic characteristics, brain metabolism exhibited a significant increase in the sACC, cOFC and bed nucleus of stria terminalis (BNST) (Fox and Kalin, 2014; Fox et al., 2015). PET imaging work with macaque monkeys has shown that the ventral pallidum (VP) is likely implicated in anxious behaviors along with coactivations of the amygdala and anterior insula (Galineau et al., 2017). These results suggest that the innate traits of anxiety disorder could be associated with the concurrent activities of those multiple brain sites. However, few studies have addressed the causal involvement of those regions in pessimistic judgment under an anxiety-like state. We here hypothesize that the primates have a “causal network for pessimistic decision-making” consisting of sites within the pACC and cOFC, which could reciprocally interact with the amygdala and other limbic sites. We further hypothesize that the primate anxiety network sends output signals to the striosome compartment of the striatum (Figure 8), which is in a position to modulate the dopaminergic system, as well as to the thalamus and the Cdt, part of an avoidance circuit. Importantly, one of the regions that exhibited the elevated activation in the AT macaque was the BNST (Fox et al., 2010, 2018), and the BNST is essential to fear responding (Grupe and Nitschke, 2013). The BNST has been shown in rodents to send a strong preferential projection to striosomes (Smith et al., 2016), supporting our hypothesis that a main downstream target of anxiety-related brain regions is the striosome compartment.

In addition to the involvement of striosomes in pessimistic decision-making, a part of the VS could also be involved in anxiety behaviors. Previous macaque studies demonstrated that the local infusion of the bicuculine to the VS induced stereotypic behavior such as licking and biting fingers (Worbe et al., 2009), suggesting that the activity originating from the VS could generate an anxiety-like behavior. The VS sends outputs to the internal segment of GP and the SNr, and to the VP and subthalamic nucleus with receiving inputs from pACC (Sgambato-Faure et al., 2016). Previous studies showed that the NAcc core lacked KChIP1 staining, similar to the low levels found in the matrix. It is not clear how the striosome-matrix delineations evident dorsally fit with those farther ventral in the caudate-putamen (e.g., dorsal-fill, ventral-avoid patterns of striosome-matrix delineations) (Graybiel and Ragsdale, 1978; Ragsdale and Graybiel, 1981). These results suggest that the anxiety-related circuitry in the VS and the NAcc core could be mediated by the circuit in the putative matrix compartment. Particularly, bicuculine infusion in the VP has been shown to induce an anxiety-like behavior such as finger biting (Galineau et al., 2017; Saga et al., 2017a, b, 2019), raising the possibility that the VS-VP pathway could be particularly important nodes for anxiety-like behaviors.

A recent series of studies using genetically engineered mice have suggested marked differences in functions of the striosome and matrix compartments in the anterior dorsomedial striatum. Optogenetically induced activation of matrix neurons elicited an increase in choices to approach large-reward outcomes neglecting their high cost (Friedman et al., 2017). Further, striosomal activity was shaped in coordination with valence-based learning, in contrast to a lack of such correlated activity in the matrix compartment (Friedman et al., 2020). Thus the striosome compartment of the striatum could be essential for learning about the values of good and bad outcomes of decisions, whereas the matrix could process the value of actions. Together with these findings, we propose that the fronto-striosomal pathway may be involved in the learning and consolidating pessimistic decision-making by modulating dopaminergic activity. Our hypothesis is consistent with the AC architecture of the striatal circuit model. The striosomes correspond to the critics, which contribute to calculating the learning signal, whereas the matrix corresponds to the actors that function to generate an action (Houk and Adams, 1995; Doya et al., 2002). We also found that microstimulation of the primate pACC (Amemori and Graybiel, 2012) and CN (Amemori et al., 2018) induced gradual change in the CBR, supporting the hypothesis that the striosome-related circuitry could be primarily involved in an adaptive change in value judgment or valence-based learning.

We previously proposed as a potential function of striosomes as the part of calculating responsibility signals in hierarchical learning (Amemori et al., 2011). In this view, the striosomal activity was hypothesized to facilitate the corresponding matrix module to be activated in the basal ganglia circuitry. Dopaminergic learning signals were locally regulated by the acetylcholine signals, resulting in the consolidation of the selected module. This view does not exclude the role of the matrix in the limbic system in the acute or chronic emergence of anxiety-like behavior. The activation of the VS/NAcc core is known to generate actions typical of anxious animals including humans (Figure 8). In our hypothesis, the striosomal circuitry could be specifically involved in facilitating the activity that could be linked to particular cortico-striato-nigral circuits and related connectivity networks. Striosomal activity could increase (or decrease) the likelihood of corresponding actions, including anxious actions, to emerge in the modular system. We note that we have not considered the likely multiple functions of the large matrix compartment of the striatum. This topic is beyond the scope of our review. But for all discussions of striosome, matrix or other subdivisions of the striatum, it is crucial to indicate the regional specializations of the striatal components, such as D1 and D2 direct and indirect pathway component functions and different distributions of other striatal molecular and connectional patterns. We also note that we have only focused here on the anterior striatum (i.e., the CN and anterior putamen in primate and the anterior dorsomedial caudoputmen in rodents).

### Pathophysiology and the Causal Network for Pessimistic Decision-Making

The symptoms of major depressive disorder (MDD) can be characterized as those showing low motivation in challenging or conflict conditions. Taking advantage of the feature that valence and arousal differentially respond to reward and punishment (Lang et al., 1998; Bradley et al., 2008; Loggia et al., 2011), approach-avoidance conflict tasks can dissociate the neuronal processes related to pessimistic judgment and motivation. Given that people with anxiety and depression differentially react to conflict conditions (Dickson and MacLeod, 2004; Dickson, 2006), the task provides a way to dissociate the biological bases of these disorders. Recently, in unmedicated patients with MDD, we observed blunted pACC responses to the avoidance condition (Ironside et al., 2020). Furthermore, the most prominent differential activities between MDD patients and healthy controls were found in the dlPFC and the NAcc. We observed the responses of the dlPFC and NAcc to the non-conflict condition were significantly reduced in MDD patients, suggesting important translational biomarkers of MDD. In the macaque experiments, we have performed characterization of the single neurons in the dlPFC and found that the dlPFC neurons were activated for the low motivational condition (Amemori et al., 2015). The blunted dlPFC activity in MDD could thus correspond to the low motivational state of the MDD patients. Similar activity patterns between dlPFC and NAcc in the MDD suggest that the NAcc could also be involved in the motivational regulation in conflict conditions, and the reduced NAcc activity observed in the MDD patients might correspond to the patient’s low motivational state. Such involvement of the VS/NAcc in motivation for both rewarding and aversive outcomes was observed in human fMRI studies (Kohls et al., 2013; Bartra et al., 2013). With these experimental findings, we here hypothesize that the dlPFC and NAcc may be involved in the motivational aspects of pessimistic decision-making.

## Conclusion

We here have reviewed studies of approach-avoidance decision-making, concentrating on non-human primate work as a window to mechanisms underlying putative proxies of pessimistic states in humans. We here propose a “causal network for pessimistic decision-making,” which we posit can produce a causal chain of influence on avoidance decision-making. Based on our new anatomical findings, in conjunction with our recording, microstimulation and anxiolytic administration findings, we suggest that avoidance behavior specifically associated with motivationally challenging decisions introduced by approach-avoidance conflict engages this network. Because one of the major output stations of the proposed pessimistic-decision network, the striosome compartment of the striatum, now has definitively been shown capable of regulating the activity of dopamine-containing SNc neurons, we emphasize that this network for pessimistic-decision could, via subsets of striosomes, regulate dopamine-related signaling responsible for, or as a modulator of, anxiety-like state.

## Data Availability Statement

The raw data supporting the conclusions of this article will be made available by the authors, without undue reservation.

## Ethics Statement

The animal study was reviewed and approved by the Committee on Animal Care of the Massachusetts Institute of Technology.

## Author Contributions

SA, K-IA, and AG designed the new experiments cited here and for these SA collected and analyzed the new data. SA, K-IA, and AG wrote the manuscript. All authors contributed to the article and approved the submitted version.

## Conflict of Interest

The authors declare that the research was conducted in the absence of any commercial or financial relationships that could be construed as a potential conflict of interest.

## Abbreviations

AP: Anterior-Posterior
GFP: Green Fluorescent Protein
MOR: μ-Opioid Receptor.
